# Postprandial glucose management of people with insulin-treated diabetes

**DOI:** 10.3389/fendo.2026.1947837

**Published:** 2026-10-23

**Authors:** Janet B. McGill, Irl B. Hirsch, Carol J. Levy, Eda Cengiz, Grazia Aleppo, Anders L. Carlson

**Affiliations:** 1 Division of Endocrinology, Metabolism & Lipid Research, Washington University, St Louis, MO, United States; 2 Division of Metabolism, Endocrinology and Nutrition, University of Washington, Seattle, WA, United States; 3 Department of Medicine, Division of Endocrinology, Diabetes and Bone Disease, Icahn School of Medicine at Mount Sinai, New York, NY, United States; 4 Department of Pediatrics, University of California, San Francisco, San Franciso, CA, United States; 5 Department of Medicine, Endocrinology, Metabolism and Molecular Medicine, Northwestern University Feinberg School of Medicine, Chicago, IL, United States; 6 International Diabetes Center, Minneapolis, MN, United States

**Keywords:** automated insulin delivery, continuous glucose monitoring, diabetes, hyperglycemia, hypoglycemia, inhaled insulin, postprandial glucose

## Abstract

Diabetes is a highly prevalent, chronic condition requiring complex treatment strategies and close monitoring. Achievement of optimal glucose management reduces vascular complications of diabetes and the long-term burden of the disease. Attenuation of postprandial rise in glucose is a critical component of overall glycemic management and often the most difficult for people with insulin-treated diabetes to achieve. While postprandial glucose (PPG) elevations are a consequence of insufficient endogenous insulin secretion or exogenous insulin dose to cover ingested carbohydrates, a mismatch between the absorption of nutrients and peak insulin concentration is also a key factor in managing PPG. Type 2 diabetes is characterized by loss of first-phase insulin secretion, resulting in elevations in PPG and prolongation of hyperglycemia after eating. In type 1 diabetes and other forms of insulin-requiring diabetes, insulin administration must match carbohydrate and other nutrient intake in both quantity and timing. Continuous glucose monitoring (CGM) has provided insights into glucose patterns and the effectiveness of diabetes therapies in managing glucose levels at all times of the day. When CGM is used with an automated insulin delivery pump or connected insulin pen, prandial insulin dosing can be analyzed for more effective coaching and education. Based on the 2026 American Diabetes Association Standards of Care guidelines, delivery devices and method of insulin administration should be assessed regularly and individualized based on the needs of each person, considering options such as automated insulin delivery, inhaled insulin, or multiple daily injections. This manuscript reviews the causes and contributors of postprandial hyperglycemia and strategies for its management. In particular, use of lifestyle management, insulin dose adjustments, timing of premeal insulin doses, and insulin kinetics will be reviewed for precision management of postprandial hyperglycemia.

## 1 Introduction

In 2023, 40.1 million people were estimated to have diagnosed and undiagnosed diabetes in the US, representing approximately 12% of the US population (1). Approximately 5% to 10% have type 1 diabetes (T1D) and 90% have type 2 diabetes (T2D) (2). Among adults living with T2D in 2022, approximately 30% required insulin therapy (3), while all individuals with T1D require physiologic replacement of insulin to cover basal and prandial needs (4).

Automated insulin delivery (AID) systems offer people with diabetes a treatment option to help improve blood glycemic outcomes; however, these devices still require input from users for bolus insulin dosing (5). Manual entry of carbohydrate intake is required for bolus insulin dosing by most AID systems (5), a task that requires significant skill on the part of the pump user for delivery of the correct dose at the optimal time to match the peak insulin concentration with nutrient absorption to avoid hyperglycemia and hypoglycemia. While a substantial percentage of individuals with T1D and a growing number of people with insulin-requiring T2D now use AID systems (5, 6), intensified insulin therapy that includes both basal and premeal insulin that is provided by injection or inhalation remains common among insulin-taking individuals (4).

The American Diabetes Association (ADA) guidelines recommend keeping hemoglobin A1C (A1C) <7% (7), whereas the American Association of Clinical Endocrinology (AACE) recommends ≤6.5% (8). Both guidelines recommend a goal of >70% of time per day within the glucose target range of 70 to 180 mg/dL (3.9 to 10 mmol/L) (7, 8). However, many individuals with insulin-treated diabetes struggle to achieve these glycemic targets (9).

Managing diabetes requires understanding the interplay among the factors that determine A1C levels, including fasting plasma glucose (FPG) and postprandial glucose (PPG) (10, 11). It has been previously suggested that FPG has a greater impact on A1C when diabetes is poorly managed, whereas PPG becomes increasingly important as patients approach A1C target values (12). However, others have noted that the nature of this relationship may be affected by the use of oral hyperglycemic drugs (13). Newer agents, including sodium-glucose cotransporter 2 (SGLT2) inhibitors, glucagon-like peptide 1 (GLP-1) receptor agonists, and dual hormone receptor agonists, have demonstrated beneficial effects on PPG (14–16). However, precise management of PPG remains problematic for many people with diabetes, especially those with insulin deficiency who require prandial insulin to achieve uptake and metabolism of ingested nutrients while avoiding hypoglycemia (11).

Historically, PPG excursions were overlooked owing to lack of routine testing capillary blood glucose after meals; however, with increasing use of continuous glucose monitoring (CGM), it is possible to evaluate both fasting and PPG values (17). CGM metrics provide retrospective information about averaging interstitial glucose levels over 24 hours but do not differentiate daytime vs nighttime or FPG vs PPG values (18). Interrogation of daily or summary glucose tracings provides insight into how well individuals are able to manage PPG (5). The question remains: how should we counsel individuals with diabetes who have significant postprandial hyperglycemia? Outside of pregnancy, guidelines recommend assessing PPG when A1C remains above target despite achieving preprandial targets, with a peak PPG goal of <180 mg/dL, with fewer specific treatment recommendations focused on PPG than in pregnancy (19). Some have recommended setting peak glucose targets of <140 to 180 mg/dL during the postprandial period, returning to preprandial values within 3 hours (7, 20). This implies that limiting the PPG rise to 60 to 80 mg/dL from preprandial values would be needed to achieve optimal glucose management. Addressing PPG rise in people who take insulin requires attention to the nutrient content of meals, accuracy of carbohydrate counting, insulin dose and timing, and consideration of faster-acting insulins (20). For individuals at high risk of hypoglycemia (eg, older adults and those with hypoglycemia unawareness), careful consideration should be given to appropriate and individualized glycemic targets and drug selection that minimizes hypoglycemic risk while effectively reducing PPG excursions (7, 21). This targeted narrative review was informed by nonsystematic searches of PubMed using combinations of terms related to postprandial glucose, diabetes, insulin therapy, continuous glucose monitoring, and relevant therapeutic approaches. Searches were supplemented by review of relevant references identified from retrieved publications and current diabetes clinical practice guidelines. Publications were selected based on their relevance to the topics addressed in this review; formal systematic-review inclusion and exclusion criteria were not applied.

## 2 Etiology of postprandial hyperglycemia

Postprandial hyperglycemia is one of the earliest glycemic abnormalities to occur in T2D (11); however, the etiology of postprandial hyperglycemia is complex, with variability in individual glycemic responses (22). Contributors to PPG rise and persistence of hyperglycemia in the postprandial period are multifactorial and vary among individuals with diabetes. Impaired functioning of beta-cells and insufficient insulin secretion is a hallmark of postprandial hyperglycemia; however, insulin resistance in the liver and peripheral tissues also contributes (23). In addition, inadequate insulin secretion from beta-cells fails to suppress production of glucagon, leading to dysregulated hepatic glucose production via increased rates of gluconeogenesis and glycogenolysis, resulting in increased release of glucose into the bloodstream (24). The adipokine leptin adds to the problem as those with T2D are “leptin resistant,” resulting in an increase in appetite in addition to an increase in dysregulation of lipid storage and an increase in hepatic glucose output (25). Together, overproduction of glucose from the liver and impaired uptake from peripheral tissues contribute greatly to the disruption of glucose homeostasis and ultimately the development of T2D (24).

The liver plays a key role in establishing postprandial glucose levels (26, 27). After a meal, the liver shifts from a fasting state of glucose production to a state of glucose absorption and storage in the form of glycogen (28). The liver is estimated to be responsible for approximately 60% to 65% of the disposal of oral glucose load (28). In individuals with chronic hyperglycemia, alterations in the metabolism of glycogen often occur, including reduced postprandial glycogen synthesis, leading to prolonged durations of hyperglycemia (28). In T2D, postprandial hyperglycemia implies that there is impaired suppression of endogenous glucose production, a primary function of insulin. Similarly in people with T1D with unmanaged PPG, the rate of endogenous glucose production suppression is increased and glycogen metabolism is altered, contributing to increased PPG (29). All of the rapid-acting insulins work by inhibiting hepatic glucose production and stimulating peripheral uptake of glucose by both fat and muscle, but onset of action and duration of effect vary among the various types of prandial insulin (5, 30, 31).

Genetic variation may contribute to interindividual differences in PPG responses through effects on intestinal glucose absorption and pathways governing insulin sensitivity and secretion (32, 33). Pharmacogenetic variants have also been associated with differential responses to glucose-lowering therapies, including metformin; however, further validation is needed before these findings can be used to inform individualized treatment selection (34). Nutritional factors also considerably impact glucose levels in diabetes, including the type and quantity of nutrients ingested, with carbohydrates having the greatest impact on PPG in most people with diabetes (35, 36). However, given the substantial variability in behaviors and glycemic response rates among individuals with diabetes, there is a continued need for personalized nutrition therapy to effectively regulate postprandial excursions (22). Availability of CGM data has allowed for an improved understanding of postprandial hyperglycemia, including the impact of carbohydrates, protein and fat consumption, and timing of meal bolus doses (20). The availability of CGM and AID system data allows healthcare professionals to evaluate the effectiveness of nutrition recommendations and insulin dosing and to identify user miscalculations based on related dose amount or timing of administration in each domain (20).

## 3 Impact of PPG in diabetes

PPG excursions may negatively affect outcomes for people with diabetes (**Figure 1**). Postprandial hyperglycemia is a direct and independent risk factor for cardiovascular disease and might exert its harmful effects through the production of free radicals (37, 38), although precise mechanisms for this physiologic effect remain unclear (39). Inadequate PPG management, commonly associated with higher A1C levels, contributes to higher glucose exposure and ultimately to microvascular (ie, nephropathy and retinopathy) and macrovascular (ie, cardiovascular disease and stroke) complications, both of which are major causes of mortality (39, 40).

**Figure 1:**
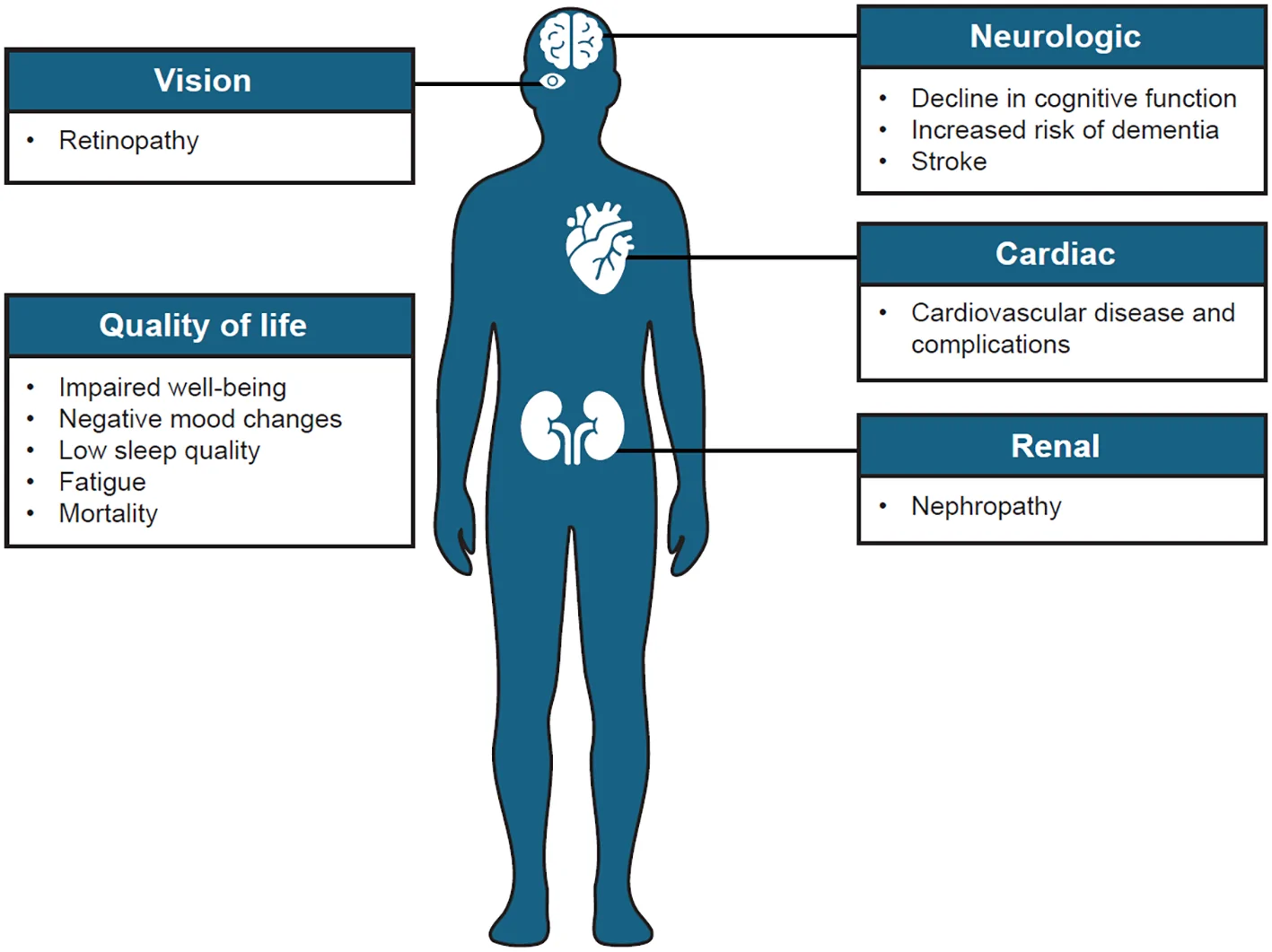
Illustration demonstrating the negative effects of postprandial glucose excursions on patients with diabetes.

In healthy individuals with normoglycemia, peak PPG often occurs approximately 30 minutes after a meal (11) and is typically <120 mg/dL and rarely >140 mg/dL (41). However, the amplitude and duration of PPG excursions is dependent on several factors mainly related to diet, including composition, quantity, and timing of meals (42–44). Considering that reducing the amplitude and duration of PPG excursions may be beneficial to long-term glycemic management and cardiovascular risk (39), managing PPG levels should be considered an integral part of diabetes management.

ADA clinical practice guidelines recommend monitoring PPG (**Table 1**) in pregnant people with diabetes and in nonpregnant adults who have not met A1C goals despite achieving preprandial glucose targets (7, 19). Per the guidelines, PPG should be measured 1 to 2 hours following the start of a meal to capture the peak level in most individuals with diabetes (7).

**Table 1:** Current guidelines for diagnosis and management of diabetes.

	ADA (7, 81)	AACE (8)	IDF^e^ (100)
Diagnosis			
A1C	≥6.5% (≥48 mmol/mol)	≥6.5%	≥6.5% (48 mmol/mol)
FPG	≥126 mg/dL^a^ (≥7.0 mmol/L)	≥126 mg/dL	≥126 mg/dL (≥7.0 mmol/L)
PPG 2-h after OGTT	≥200 mg/dL (≥11.1 mmol/L)	≥200 mg/dL	≥200 mg/dL (≥11.1 mmol/L)
Glycemic management			
A1C	<7% (<53 mmol/mol) for nonpregnant adults	≤6.5%^d^	<7% (<53 mmol/mol)
FPG	80–130 mg/dL^b^ (4.4-7.2 mmol/L)	<110 mg/dL	<110 mg/dL (<6 mmol/L)
PPG (2-h)	<180 mg/dL^c^ (<10.0 mmol/L)	<140 mg/dL	<180 mg/dL (<10 mol/L)
TIR/TBR	70–180 mg/dL: >70% <70 mg/dL mg/dL: <4% <54 mg/dL: <1%	70–180 mg/dL: >70% <70 mg/dL: <4% <54 mg/dL: <1%	–

A1C, hemoglobin A1C; AACE, American Association of Clinical Endocrinology; ADA, American Diabetes Association; FPG, fasting plasma glucose; IDF, International Diabetes Federation; OGTT, oral glucose tolerance test; PPG, postprandial glucose; TBR, time below range; TIR, time in range. ^a^Fasting is defined as no caloric intake for ≥8 hours. ^b^For healthy individuals (few coexisting chronic illnesses, intact cognitive and functional status). ^c^More or less stringent glycemic goals may be appropriate for certain individuals. ^d^Adopt less stringent glycemic goals (A1C 7%-8%) in persons with a history of severe hypoglycemia, hypoglycemia unawareness, limited life expectancy, advanced renal disease, extensive comorbid conditions, or long-standing diabetes in which the A1C goal has been difficult to attain despite intensive efforts, so long as the person remains free of hyperglycemia-associated symptoms. ^e^Diagnoses and guidelines for type 2 diabetes.

## 4 Methods for measuring PPG

### 4.1 CGM has simplified the overall evaluation of multiple measures of glycemic management

With the introduction of CGM, evaluation of PPG can be done by inspection of daily or composite glucose patterns, along with time in range metrics (17, 45). Time in tight range serves as a glycemic metric that reflects normal glucose patterns seen in healthy individuals, measured by the percentage of time glucose falls within 70 to 140 mg/dL (46). One study using CGM in individuals without diabetes found a median of 96% time in tight range (47), supporting the notion that FPG should be close to 100 mg/dL and PPG should be <140 mg/dL to achieve glucose levels near normal ranges. PPG elevations have been observed in about 39% of patients with optimal A1C values (48, 49). A 2-hour PPG goal of <180 mg/dL has been associated with an A1C of ≤7% (7), but this may not hold true in all individual patients (48, 50). AACE has recommended a 2-hour PPG goal of <140 mg/dL to achieve an A1C level of ≤6.5% (8).

Interestingly, a peak in PPG is reached at approximately 30 minutes after the start of a meal in individuals with normal glucose tolerance, >30 minutes in individuals with impaired glucose tolerance, and >60 minutes in individuals with T2D (11, 51–53). The rightward shift in the timing of peak PPG reflects reduced first-phase insulin secretion and reduced incretin effect contributing to lower prandial insulin secretion, leading to ineffective management of hepatic glucose output and less efficient muscle glucose uptake (54).

Reports provided by CGM-connected systems also provide insight into common behavior pitfalls that impact postprandial glucose, including delayed or missed bolus insulin dosing, imprecise carbohydrate counting, and reliance on postmeal corrections (20), which may lead to high postprandial peak glucose, larger area under the glucose curve, and later postprandial hypoglycemia due to insulin dose stacking. CGM identifies the glucose rise with nutrient ingestion, including the peak glucose level and duration of postprandial hyperglycemia (55). Examination of AID pumps and connected insulin pen downloads provides insight into patient prandial dosing behaviors and adequacy of preset insulin-to-carbohydrate ratios (5, 56). Achieving recommended PPG targets requires accurate estimation of the nutrient content of meals or snacks, particularly carbohydrate content, and appropriate timing of the premeal insulin dose (57). Despite these advancements in technology and reporting systems, inconsistencies and miscalculations are still common owing to factors such as patient error, food composition, trend arrows, medications, exercise, and environmental considerations (20).

## 5 Achieving glycemic targets through lifestyle modifications and medication

### 5.1 Lifestyle modifications

The ADA recommends lifestyle modifications such as nutritional therapy, weight management, and physical activity, to all patients with diabetes to aid diabetes management (58). Regular aerobic exercise improves glycemic management in adults with T2D, resulting in less daily time in hyperglycemia and reductions in A1C (59). The glycemic effects can be maximized by engaging in exercise in the postprandial period (59). Furthermore, eating carbohydrates last in a meal (after eating protein and/or vegetables first) may aid in regulating PPG and lowering glucose excursions (60–62).

As previously stated, carbohydrates are well established as the primary nutrient affecting PPG (35, 36). While there is no specific guideline-based recommendation from the ADA regarding what percentage of daily food intake should consist of carbohydrates, ranges of 40% to 60% have been proposed (63). Exact carbohydrate counting involves calculating each bolus insulin dose based on the grams of carbohydrates consumed per meal using an insulin-to-carbohydrate ratio, which represents the amount of carbohydrates needed to match the blood glucose-lowering effect of 1 unit of rapid-acting insulin (64). One study with 40 participants with T1D demonstrated improvements in PPG after implementation of carbohydrate counting (peak glucose 149 mg/dL) vs before carbohydrate counting (peak glucose 241 mg/dL; *P* = 0.001) (65). Limitations of traditional carbohydrate counting include the potential for human error (eg, miscalculations) that may lead to over- or underdosing of insulin (35) and the burden associated with manually counting carbohydrates (66). In the same study of patients with T1D, improvements were observed in median A1C values with flexible insulin (7.3%) vs standard insulin (8.0%) dosing regiments (*P* = 0.007) (65). Use of carbohydrate counting enables individuals with diabetes to implement flexible insulin dosing, allowing for improved glycemic management without increasing the risk of hypoglycemia (65). Flexible dosing involves a more simplified approach to dosing in which meal size (eg, small, usual, large) and premeal glucose are used to adjust bolus doses, rather than basing the dose off of the exact number of carbohydrate grams in the meal (67).

In addition to carbohydrates, protein and fat content can impact PPG levels (54, 68). Protein intake contributes to enhanced incretin effects and increased secretion of insulin, thus improving PPG (68). Consumption of fats alongside carbohydrates has demonstrated slower gastric emptying, which in turn delays the release of glucose into the blood (54). Factors such as order of macronutrient consumption, limiting amount of carbohydrates, and increased consumption of dietary proteins have demonstrated promising effects on PPG levels (68). Therefore, clinicians and people with diabetes must consider the overall meal composition when determining insulin dosing.

### 5.2 Noninsulin medications

Noninsulin medications that work to lower blood glucose through differing mechanisms are available for treatment of T2D; these include amylin analogs, alpha-glucosidase inhibitors, GLP-1 receptor agonists, SGLT2 inhibitors, meglitinides, and sulfonylureas (**Table 2**) (15, 59, 69–71). Alpha-glucosidase inhibitors improve glycemic variability by delaying the rate at which carbohydrates are absorbed and reducing PPG excursions (59, 72). GLP-1 receptor agonists improve glycemic management by augmenting insulin secretion in a glucose-dependent manner, suppressing glucagon secretion at hyperglycemia or euglycemia, slowing gastric emptying (thereby preventing large postprandial excursions), and assisting people with dietary management by reducing caloric intake (70, 73). SGLT2 inhibitors reduce plasma glucose concentrations by increasing urinary glucose excretion and often result in improved A1C levels (69, 74, 75). These newer noninsulin antihyperglycemia agents, such as the incretin mimetics and SGLT2 inhibitors, are particularly effective at lowering PPG without causing hypoglycemia, thus increasing numbers of individuals with T2D are achieving both FPG and PPG targets with these agents (74, 76, 77). Meglitinides and sulfonylureas are additional classes of noninsulin glucose-lowering agents; these short-acting oral medications stimulate insulin secretion and target PPG control (15). Compared to SGLT2 inhibitors and GLP-1 receptor agonists, meglitinides and sulfonylureas are more likely to cause hypoglycemia (78).

**Table 2:** Relative comparison of noninsulin medications used for PPG management.

Class	Effect on PPG (101–103)	Effect on FPG (101–103)	High hypoglycemia risk (4, 104)	Cost (4)	Common adverse effects (105, 106)
SGLT2 inhibitors	Moderate	Moderate	No	Moderate/high	Vulvovaginal candidiasis, urinary tract infections, hypotension, dehydration
GLP-1 receptor agonists	High	Moderate	No	High	Nausea, vomiting, diarrhea
Meglitinides	High	Moderate	Yes	Moderate/high	Weight gain
Sulfonylureas	Low	High	Yes	Low	Weight gain, dizziness, headache
Alpha-glucosidase inhibitors	High	Low	No	Moderate	Bloating, flatulence, diarrhea
Amylin analogs	High	Low	Yes	High	Nausea

FPG, fasting plasma glucose; GLP-1, glucagon-like peptide 1; PPG, postprandial glucose; SGLT2, sodium-glucose cotransporter 2. Effects have been generalized by class. Note that specific effects vary by agent.

### 5.3 Insulin and insulin analog therapy

Insulin treatment is recommended at the time of diagnosis in T2D when A1C level is >10%, blood glucose is ≥300 mg/dL, or a diagnosis of T1D is possible and other oral agents are insufficient to lower the A1C levels (4, 8, 79). Insulin treatment should also be considered in patients with undiagnosed diabetes until the distinction can be made between T1D, T2D, or an atypical form of diabetes (eg, pancreatic, maturity-onset diabetes of the young, lipodystrophy) (79). Approximately 3% to 5% of people with T2D have absolute insulin deficiency (80, 81), while most will have progressive loss of beta-cell function during their lifetime with diabetes (82). Thus, insulin therapy is needed for all patients with T1D and a portion of those with T2D and may also be required in individuals with pancreatic diabetes (4). Nearly all individuals who require insulin will need basal insulin, with intermediate-acting, long-acting, and ultra-long-acting insulins available to cover individual needs (79). People with T1D and many people with T2D or other forms of diabetes require both basal and bolus insulin (4, 79). Mealtime insulin dosing is often the most challenging aspect of diabetes therapy for individuals who require physiologic insulin replacement that includes both basal and prandial insulin needs (83).

Basal insulin doses generally comprise up to 50% of the total daily insulin requirement, adjusted to manage FPG without causing overnight hypoglycemia (4). In individuals whose diabetes is characterized by insulin deficiency, basal insulin is not sufficient to counter the mealtime rise in glucose (84). Prandial insulin, either endogenous or exogenous, is needed to inhibit hepatic glucose production and glucagon secretion from the alpha-cells of the pancreas, block the formation and release of free fatty acids from adipose tissues, and regulate hepatic glucose production via actions of insulin in the hypothalamus (85), thus managing the rise in glucose that occurs after ingestion of carbohydrates or mixed meals.

Effective use of mealtime insulin includes dosing adjustments based on anticipated carbohydrate intake along with ambient glucose levels (4). Short-acting injectable insulin has an action onset of 30 to 60 minutes with its peak glucose-lowering action occurring between 80 and 120 minutes after injection (79, 86). Injectable rapid-acting analog and ultra-rapid-acting insulin formulations were developed to accelerate absorption and the onset and duration of glucose-lowering effects (4). Rapid-acting analogs can take up to 30 minutes to have a measurable effect and up to 1 hour to reach maximum concentrations and often remain in the system for up to 5 hours (87) (**Figure 2**). While currently available subcutaneously injected rapid-acting analog formulations cause less hypoglycemia than human insulin in T1D (4), insulin may need to be administered 15 to 20 minutes before a meal to control for delays in subcutaneous insulin onset of action (87, 88); earlier administration may be needed for premeal hyperglycemia or an uptrending arrow on CGM. Additionally, lipohypertrophy, caused by repeated injections into the same skin region, can result in reduced rate and amount of insulin absorption, leading to deterioration of postprandial blood glucose management (89). To reduce risk of lipohypertrophy, education on injection/pump site rotation techniques should be reinforced (89).

**Figure 2:**
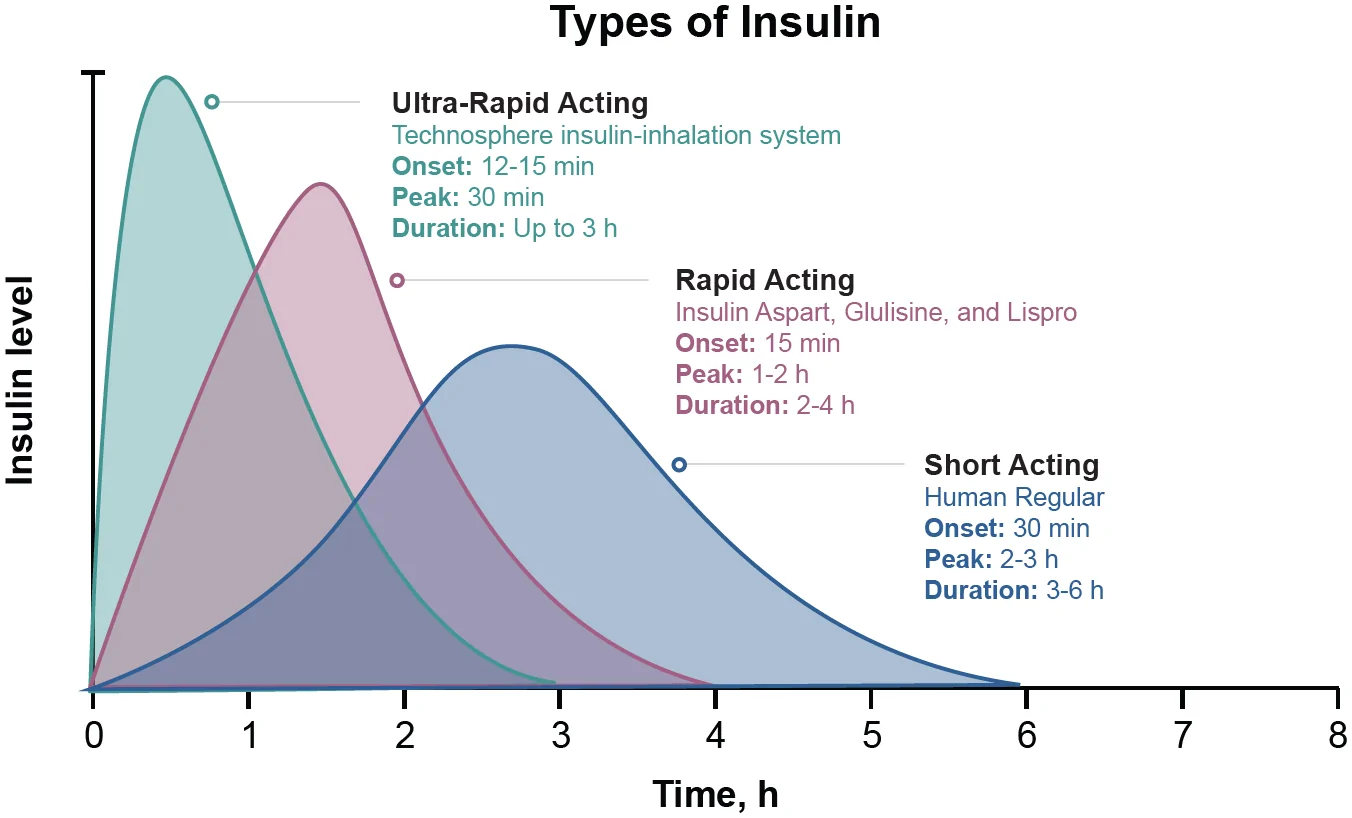
Activity profiles of ultra-rapid-, rapid-, and short-acting insulins.

Technosphere insulin (inhaled insulin) provides an alternate method of delivery of mealtime insulin and is characterized by an ultra-fast onset of action and shorter duration (4, 90). The fast onset and elimination rates help concentrate insulin effects for approximately 3 hours after a meal, allowing for improved postprandial glycemic management and more flexibility of doses (**Figure 2**) (87, 90). PPG levels were lower without increased risk of hypoglycemia in individuals with T1D using inhaled insulin compared with injected rapid-acting insulin (87). Long-acting insulin is required in individuals with T1D, and effective use of either basal-bolus therapy or continuous subcutaneous insulin infusion (or insulin pump) has been shown to reduce diabetes complications compared to less intensive approaches (91). Further, compared to subcutaneously injected rapid-acting insulin analogs, the time-action profile of inhaled insulin more closely resembles the physiologic release of prandial endogenous insulin (92), as observed in individuals without diabetes. However, inhaled insulin has several important limitations. It is contraindicated in individuals with chronic lung disease (eg, asthma or chronic obstructive pulmonary disease) because of risk of acute bronchospasm, and should be used with caution in individuals with active lung cancer, history of lung cancer, or in patients at risk for lung cancer (30). Before treatment, pulmonary function testing is required before initiation of inhaled insulin, again after 6 months of treatment, and annually thereafter to monitor pulmonary function (30). Cost and access should also be considered. An earlier economic analysis found that Exubera was more costly than subcutaneous insulin; however, this historical finding may not reflect the current cost or insurance coverage of Technosphere insulin (93).

Continuous subcutaneous insulin infusion therapy is recommended for patients with T1D as it mimics normal insulin secretion by continuously providing rapid-acting insulin with patient-activated bolus doses at mealtimes or for corrections (4, 20). It provides an alternative approach to conventional multiple daily injections (79). AID systems are currently the primary method of continuous insulin delivery in T1D owing to advantages in algorithms to modulate insulin delivery to minimize hyperglycemia and hypoglycemia (5). These systems include an insulin pump, integrated CGM, and an algorithm that mimics natural insulin replacement. AID systems are guideline recommended and should now be offered to the majority of patients with T1D and T2D as the preferred method of insulin delivery (5). AID strategies relevant to PPG management differ in the degree of user input required (94). Hybrid closed-loop systems generally require a user-initiated, carbohydrate-matched premeal bolus. Although these systems can improve overall time in range and reduce time in hypoglycemia compared with conventional insulin pump therapy, postprandial control may remain suboptimal and the need for carbohydrate counting persists. Simplified or adaptive meal-priming approaches estimate premeal insulin using meal size or type, individualized insulin-to-carbohydrate ratios, and/or insulin requirements from previous similar meals, thereby reducing the burden of precise carbohydrate counting. Fully automated approaches seek to manage unannounced meals using reactive or meal-detection algorithms without manual meal input; however, these approaches have not consistently achieved postprandial control comparable with carbohydrate-matched bolusing and may be limited by early postprandial hyperglycemia and late hypoglycemia. Multi-hormone systems represent another approach to PPG management. Insulin–glucagon systems use intermittent glucagon delivery to prevent or treat late postprandial hypoglycemia and may permit more aggressive insulin delivery early after meals. Insulin–pramlintide approaches aim to reduce PPG excursions by slowing gastric emptying and suppressing postprandial glucagon release, potentially allowing more time for the AID algorithm to adjust insulin delivery. However, these approaches have distinct technical and safety limitations that require further evaluation (94).

Based on the most recent 2026 ADA guidelines, adequacy of insulin dose and therapeutic management should be assessed at every clinician visit (4). Insulin needs should be individualized and method of administration should be modified as needed to limit glucose variability (eg, switch between multiple daily injections, AID, or inhaled insulin) (4).

### 5.4 Understanding the risk of hypoglycemia

Hypoglycemia is a potential risk when attempting to tightly manage PPG (95). Incidence of severe hypoglycemia has been reported in 36 to 41 episodes per 100 patient-years in people with T1D (96) and 35 episodes per 100 patient-years in people with T2D (97). Severe hypoglycemia in patients is rare, but fear of hypoglycemia remains a major obstacle that necessitates education, especially when trying to achieve tight PPG management as the fear may lower insulin-taking habits or medication taking for recommended therapies and set up cycles of higher doses of insulin followed by “defensive eating” and weight gain (20, 98). Select populations at high risk for hypoglycemia should have individualized PPG goals and may require more frequent monitoring (7). For example, in older adults, glycemic targets should account for factors such as cognitive and functional status, frailty, comorbidities, life expectancy, meal regularity, and treatment burden, with less stringent goals where appropriate (7, 21). In individuals with impaired hypoglycemia awareness or recurrent clinically significant hypoglycemia, avoidance of further hypoglycemia should take priority, with reassessment of the treatment regimen and deintensification where appropriate to reduce hypoglycemia risk (7). Lower rates of hypoglycemia have been reported with inhaled insulin compared with rapid-acting subcutaneous insulin (87, 90, 99). One study demonstrated higher rates of hypoglycemia 30–60 minutes after meals with inhaled insulin vs insulin aspart, but significantly lower rates 2–6 hours after meals (90). Another study demonstrated significantly lower rates of mild and moderate hypoglycemia event rates with inhaled insulin in the first 90 minutes postmeal compared to insulin lispro (99). Further, CGM can help to eliminate concerns of overdosing insulin, which may lead to hypoglycemia (20).

## 6 Discussion

Postprandial hyperglycemia is a direct and independent risk factor for microvascular complications of diabetes and for cardiovascular disease. Currently, the ADA recommends peak PPG <180 mg/dL, measured 1–2 hours after meal initiation, whereas the AACE algorithm notes that achieving an individualized A1C goal of ≤6.5% may require 2-hour PPG <140 mg/dL (7, 8). Both guidelines align with findings of this review by recommending individualized diabetes treatment, including optionality of a variety of insulin delivery methods (eg, multiple daily injections, AID, and inhaled insulin) to reduce glucose variability. Further, the use of CGM with time in range and time in tight range targets can improve PPG levels and overall diabetes outcomes. Monitoring and addressing PPG excursions through meal composition, insulin type, timing, dosing, and exercise may improve glycemic management in people with diabetes. Additional studies using CGM to target PPG are warranted to support the implementation of PPG as an important indicator of diabetes management.

## References

[1] Centers for Disease Control and Prevention . National Diabetes Statistics Report. (2026).

[2] Goyal R Singhal M Jialal I . Type 2 diabetes. In: Statpearls (Internet). Treasure Island, FL (2023).

[3] Hou W Tuttle KR Shen W Reikes A Watanabe JH . Trends in pharmacological treatment of patients with new onset type 2 diabetes: usage patterns in an evolving guideline landscape. J Diabetes. (2025) 17:e70108. doi: 10.1111/1753-0407.70108 40464139 PMC12134773

[4] American Diabetes Association . 9. Pharmacologic approaches to glycemic treatment: Standards of Care in Diabetes-2026. Diabetes Care. (2026) 49:S183–215. doi: 10.2337/dc26-S009 41358900 PMC12690185

[5] American Diabetes Association . 7. Diabetes technology: standards of care in diabetes-2026. Diabetes Care. (2026) 49:S150–65. doi: 10.2337/dc26-S007 41358889 PMC12690173

[6] Zeng B Jia H Yang S Zheng J Yang Q Dou L . Effectiveness and safety of automated insulin delivery in type 2 diabetes: a meta-analysis. Diabetol Metab Syndr. (2025) 17:433. doi: 10.1186/s13098-025-02014-x 41254762 PMC12624994

[7] American Diabetes Association . 6. Glycemic goals, hypoglycemia, and hyperglycemic crises: Standards of Care in Diabetes-2026. Diabetes Care. (2026) 49:S132–49. doi: 10.2337/dc26-S006 41358894 PMC12690178

[8] Samson SL Vellanki P Blonde L Hirsch IB Hoang TD Isaacs SD . American Association of Clinical Endocrinology consensus statement: algorithm for management of adults with type 2 diabetes - 2026 update. Endocr Pract. (2026) 32:473–518. doi: 10.1016/j.eprac.2026.01.006 41842862

[9] Foster NC Beck RW Miller KM Clements MA Rickels MR DiMeglio LA . State of type 1 diabetes management and outcomes from the T1D exchange in 2016-2018. Diabetes Technol Ther. (2019) 21:66–72. doi: 10.1089/dia.2018.0384 30657336 PMC7061293

[10] Ceriello A . The glucose triad and its role in comprehensive glycaemic control: current status, future management. Int J Clin Pract. (2010) 64:1705–11. doi: 10.1111/j.1742-1241.2010.02517.x 20860758 PMC3036818

[11] Joshi S Kesavadev J K MP Saboo B Mehta A Bhattacharyya A . Postprandial glucose: a variable in continuum. Clin Med Insights Endocrinol Diabetes. (2025) 18:11795514251370507. doi: 10.1177/11795514251370507 41030889 PMC12477350

[12] Monnier L Lapinski H Colette C . Contributions of fasting and postprandial plasma glucose increments to the overall diurnal hyperglycemia of type 2 diabetic patients: variations with increasing levels of HbA(1c). Diabetes Care. (2003) 26:881–5. doi: 10.2337/diacare.26.3.881 12610053

[13] Ramachandran A Riddle MC Kabali C Gerstein HC . Relationship between A1C and fasting plasma glucose in dysglycemia or type 2 diabetes: an analysis of baseline data from the ORIGIN trial. Diabetes Care. (2012) 35:749–53. doi: 10.2337/dc11-1918 22323416 PMC3308289

[14] Padda IS Mahtani AU Parmar M . Sodium-glucose transport 2 (SGLT2) inhibitors. In: Statpearls (Internet). Treasure Island, FL (2026). 35015430

[15] Owens DR Monnier L Hanefeld M . A review of glucagon-like peptide-1 receptor agonists and their effects on lowering postprandial plasma glucose and cardiovascular outcomes in the treatment of type 2 diabetes mellitus. Diabetes Obes Metab. (2017) 19:1645–54. doi: 10.1111/dom.12998 28474401 PMC5697665

[16] Coskun T Sloop KW Loghin C Alsina-Fernandez J Urva S Bokvist KB . LY3298176, a novel dual GIP and GLP-1 receptor agonist for the treatment of type 2 diabetes mellitus: From discovery to clinical proof of concept. Mol Metab. (2018) 18:3–14. doi: 10.1016/j.molmet.2018.09.009 30473097 PMC6308032

[17] Battelino T Danne T Bergenstal RM Amiel SA Beck R Biester T . Clinical targets for continuous glucose monitoring data interpretation: recommendations from the International Consensus on Time in Range. Diabetes Care. (2019) 42:1593–603. doi: 10.2337/dci19-0028 31177185 PMC6973648

[18] Martens TW Simonson GD Bergenstal RM . Using continuous glucose monitoring data in daily clinical practice. Cleve Clin J Med. (2024) 91:611–20. doi: 10.3949/ccjm.91a.23090 39353661

[19] American Diabetes Association . 15. Management of diabetes in pregnancy: Standards of Care in Diabetes-2026. Diabetes Care. (2026) 49:S321–38. doi: 10.2337/dc26-S015 41358885 PMC12690181

[20] Leahy JJL Aleppo G Fonseca VA Garg SK Hirsch IB McCall AL . Optimizing postprandial glucose management in adults with insulin-requiring diabetes: report and recommendations. J Endocr Soc. (2019) 3:1942–57. doi: 10.1210/js.2019-00222 31608313 PMC6781941

[21] American Diabetes Association . 13. Older adults: standards of care in diabetes-2026. Diabetes Care. (2026) 49:S277–96. doi: 10.2337/dc26-S013 41358888 PMC12690186

[22] Brugger V Kowatsch T Jovanova M . Predicting postprandial glucose excursions to personalize dietary interventions for type-2 diabetes management. Sci Rep. (2025) 15:25920. doi: 10.1038/s41598-025-08003-4 40676054 PMC12271334

[23] Wang L Li L Liu J Sheng C Yang M Hu Z . Associated factors and principal pathophysiological mechanisms of type 2 diabetes mellitus. Front Endocrinol (Lausanne). (2025) 16:1499565. doi: 10.3389/fendo.2025.1499565 40416523 PMC12098035

[24] Rines AK Sharabi K Tavares CD Puigserver P . Targeting hepatic glucose metabolism in the treatment of type 2 diabetes. Nat Rev Drug Discov. (2016) 15:786–804. doi: 10.1038/nrd.2016.151 27516169 PMC5751421

[25] Salazar J Chavez-Castillo M Rojas J Ortega A Nava M Perez J . Is "leptin resistance" another key resistance to manage type 2 diabetes? Curr Diabetes Rev. (2020) 16:733–49. doi: 10.2174/1573399816666191230111838 31886750

[26] Sherwin RS . Role of the liver in glucose homeostasis. Diabetes Care. (1980) 3:261–5. doi: 10.2337/diacare.3.2.261 6993137

[27] Cherrington AD . Banting Lecture 1997. Control of glucose uptake and release by the liver *in vivo*. Diabetes. (1999) 48:1198–214. doi: 10.2337/diabetes.48.5.1198 10331429

[28] Moore MC Coate KC Winnick JJ An Z Cherrington AD . Regulation of hepatic glucose uptake and storage *in vivo*. Adv Nutr. (2012) 3:286–94. doi: 10.3945/an.112.002089 22585902 PMC3649460

[29] Bischof MG Bernroider E Krssak M Krebs M Stingl H Nowotny P . Hepatic glycogen metabolism in type 1 diabetes after long-term near normoglycemia. Diabetes. (2002) 51:49–54. doi: 10.2337/diabetes.51.1.49 11756322

[30] MannKind Corporation . Afrezza® (insulin human inhalation powder, for oral inhalation use). Full Prescribing Information. Westlake Village, CA: MannKind Corporation (2026).

[31] Mehta RJ Gastaldelli A Balas B Ricotti A DeFronzo RA Tripathy D . Mechanism of action of inhaled insulin on whole body glucose metabolism in subjects with type 2 diabetes mellitus. Int J Mol Sci. (2019) 20:4230. doi: 10.3390/ijms20174230 31470605 PMC6747203

[32] Peschard S Raverdy V Bauvin P Goutchtat R Touche V Derudas B . Genetic evidence of causal relation between intestinal glucose absorption and early postprandial glucose response: A mendelian randomization study. Diabetes. (2024) 73:983–92. doi: 10.2337/db23-0805 38498375 PMC11109783

[33] Kahn SE Suvag S Wright LA Utzschneider KM . Interactions between genetic background, insulin resistance and beta-cell function. Diabetes Obes Metab. (2012) 14 Suppl 3:46–56. doi: 10.1111/j.1463-1326.2012.01650.x 22928564 PMC3634618

[34] Jablonski KA McAteer JB de Bakker PI Franks PW Pollin TI Hanson RL . Common variants in 40 genes assessed for diabetes incidence and response to metformin and lifestyle intervention in the diabetes prevention program. Diabetes. (2010) 59:2672–81. doi: 10.2337/db10-0543 20682687 PMC3279522

[35] Amorim D Miranda F Santos A Graca L Rodrigues J Rocha M . Assessing carbohydrate counting accuracy: current limitations and future directions. Nutrients. (2024) 16:2183. doi: 10.3390/nu16142183 39064626 PMC11279647

[36] Tascini G Berioli MG Cerquiglini L Santi E Mancini G Rogari F . Carbohydrate counting in children and adolescents with type 1 diabetes. Nutrients. (2018) 10:109. doi: 10.3390/nu10010109 29361766 PMC5793337

[37] Papachristoforou E Lambadiari V Maratou E Makrilakis K . Association of glycemic indices (hyperglycemia, glucose variability, and hypoglycemia) with oxidative stress and diabetic complications. J Diabetes Res. (2020) 2020:7489795. doi: 10.1155/2020/7489795 33123598 PMC7585656

[38] Bonora E Muggeo M . Postprandial blood glucose as a risk factor for cardiovascular disease in type II diabetes: the epidemiological evidence. Diabetologia. (2001) 44:2107–14. doi: 10.1007/s001250100020 11793012

[39] Hanssen NMJ Kraakman MJ Flynn MC Nagareddy PR Schalkwijk CG Murphy AJ . Postprandial glucose spikes, an important contributor to cardiovascular disease in diabetes? Front Cardiovasc Med. (2020) 7:570553. doi: 10.3389/fcvm.2020.570553 33195459 PMC7530333

[40] Gallwitz B . Implications of postprandial glucose and weight control in people with type 2 diabetes: understanding and implementing the International Diabetes Federation guidelines. Diabetes Care. (2009) 32:S322–5. doi: 10.2337/dc09-S331 19875573 PMC2811482

[41] Parkin CG Brooks N . Postprandial glucose control important? Is it practical in primary care settings? Clin Diabetes. (2002) 20:71–6. doi: 10.2337/diaclin.20.2.71

[42] Paterson MA King BR Smart CEM Smith T Rafferty J Lopez PE . Impact of dietary protein on postprandial glycaemic control and insulin requirements in type 1 diabetes: a systematic review. Diabetes Med. (2019) 36:1585–99. doi: 10.1111/dme.14119 31454430

[43] Timmer R Bogaardt L Brummelhuis WJ van Oostrom CT van Kerkhof LWM Wong A . A randomized crossover trial assessing time of day snack consumption and resulting postprandial glycemic response in a real-life setting among healthy adults. Chronobiol Int. (2022) 39:1329–39. doi: 10.1080/07420528.2022.2105230 36004426 PMC11332402

[44] Wang B Smyl C Chen CY Li XY Huang W Zhang HM . Suppression of postprandial blood glucose fluctuations by a low-carbohydrate, high-protein, and high-omega-3 diet via inhibition of gluconeogenesis. Int J Mol Sci. (2018) 19:1823. doi: 10.3390/ijms19071823 29933567 PMC6073393

[45] Dunn TC Ajjan RA Bergenstal RM Xu Y . Is it time to move beyond TIR to TITR? Real-world data from over 20,000 users of continuous glucose monitoring in patients with type 1 and type 2 diabetes. Diabetes Technol Ther. (2024) 26:203–10. doi: 10.1089/dia.2023.0565 38444315 PMC10877396

[46] Kwon SY Moon JS . Advances in continuous glucose monitoring: clinical applications. Endocrinol Metab (Seoul). (2025) 40:161–73. doi: 10.3803/EnM.2025.2370 40195726 PMC12061739

[47] Shah VN DuBose SN Li Z Beck RW Peters AL Weinstock RS . Continuous glucose monitoring profiles in healthy nondiabetic participants: a multicenter prospective study. J Clin Endocrinol Metab. (2019) 104:4356–64. doi: 10.1210/jc.2018-02763 31127824 PMC7296129

[48] Schrot RJ . Targeting plasma glucose: preprandial versus postprandial. Clin Diabetes. (2004) 22:169–72. doi: 10.2337/diaclin.22.4.169

[49] Erlinger TP Brancati FL . Postchallenge hyperglycemia in a national sample of U.S. adults with type 2 diabetes. Diabetes Care. (2001) 24:1734–8. doi: 10.2337/diacare.24.10.1734 11574434

[50] Buse JB . Should postprandial glucose be routinely measured and treated to a particular target? No! Diabetes Care. (2003) 26:1615–8. doi: 10.2337/diacare.26.5.1615 12716828

[51] Wang X Zhao X Zhou R Gu Y Zhu X Tang Z . Delay in glucose peak time during the oral glucose tolerance test as an indicator of insulin resistance and insulin secretion in type 2 diabetes patients. J Diabetes Investig. (2018) 9:1288–95. doi: 10.1111/jdi.12834 29537733 PMC6215929

[52] Suzuki K Watanabe K Futami-Suda S Yano H Motoyama M Matsumura N . The effects of postprandial glucose and insulin levels on postprandial endothelial function in subjects with normal glucose tolerance. Cardiovasc Diabetol. (2012) 11:98. doi: 10.1186/1475-2840-11-98 22891922 PMC3471039

[53] Chung ST Ha J Onuzuruike AU Kasturi K Galvan-De La Cruz M Bingham BA . Time to glucose peak during an oral glucose tolerance test identifies prediabetes risk. Clin Endocrinol (Oxf). (2017) 87:484–91. doi: 10.1111/cen.13416 28681942 PMC5658251

[54] Blaak EE Antoine JM Benton D Bjorck I Bozzetto L Brouns F . Impact of postprandial glycaemia on health and prevention of disease. Obes Rev. (2012) 13:923–84. doi: 10.1111/j.1467-789X.2012.01011.x 22780564 PMC3494382

[55] Röhling M Martin T Wonnemann M Kragl M Klein HH Heinemann L . Determination of postprandial glycemic responses by continuous glucose monitoring in a real-world setting. Nutrients. (2019) 11:2305. doi: 10.3390/nu11102305 31569815 PMC6835966

[56] Considine EG Sherr JL . Real-world evidence of automated insulin delivery system use. Diabetes Technol Ther. (2024) 26:53–65. doi: 10.1089/dia.2023.0442 38377315 PMC10890954

[57] Krzymien J Ladyzynski P . Insulin in type 1 and type 2 diabetes-should the dose of insulin before a meal be based on glycemia or meal content? Nutrients. (2019) 11:607. doi: 10.3390/nu11030607 30871141 PMC6471836

[58] American Diabetes Association . 3. Prevention or delay of diabetes and associated comorbidities: Standards of Care in Diabetes-2026. Diabetes Care. (2026) 49:S50–60. doi: 10.2337/dc26-S003 41358891 PMC12690170

[59] Davies MJ Aroda VR Collins BS Gabbay RA Green J Maruthur NM . Management of hyperglycaemia in type 2 diabetes, 2022. A consensus report by the American Diabetes Association (ADA) and the European Association for the Study of Diabetes (EASD). Diabetologia. (2022) 65:1925–66. doi: 10.1007/s00125-022-05787-2 36151309 PMC9510507

[60] Imai S Fukui M Ozasa N Ozeki T Kurokawa M Komatsu T . Eating vegetables before carbohydrates improves postprandial glucose excursions. Diabetes Med. (2013) 30:370–2. doi: 10.1111/dme.12073 23167256 PMC3674531

[61] Shukla AP Dickison M Coughlin N Karan A Mauer E Truong W . The impact of food order on postprandial glycaemic excursions in prediabetes. Diabetes Obes Metab. (2019) 21:377–81. doi: 10.1111/dom.13503 30101510 PMC7398578

[62] Shukla AP Andono J Touhamy SH Casper A Iliescu RG Mauer E . Carbohydrate-last meal pattern lowers postprandial glucose and insulin excursions in type 2 diabetes. BMJ Open Diabetes Res Care. (2017) 5:e000440. doi: 10.1136/bmjdrc-2017-000440 28989726 PMC5604719

[63] Koloverou E Panagiotakos DB . Macronutrient composition and management of non-insulin-dependent diabetes mellitus (NIDDM): a new paradigm for individualized nutritional therapy in diabetes patients. Rev Diabetes Stud. (2016) 13:6–16. doi: 10.1900/RDS.2016.13.6 27563693 PMC5291179

[64] Schmidt S Norgaard K . Bolus calculators. J Diabetes Sci Technol. (2014) 8:1035–41. doi: 10.1177/1932296814532906 24876436 PMC4455388

[65] Gokosmanoglu F Onmez A . Influence of flexible insulin dosing with carbohydrate counting method on metabolic and clinical parameters in type 1 diabetes patients. Open Access Maced J Med Sci. (2018) 6:1431–4. doi: 10.3889/oamjms.2018.256 30159070 PMC6108809

[66] Haidar A Legault L Raffray M Gouchie-Provencher N Jafar A Devaux M . A randomized crossover trial to compare automated insulin delivery (the artificial pancreas) with carbohydrate counting or simplified qualitative meal-size estimation in type 1 diabetes. Diabetes Care. (2023) 46:1372–8. doi: 10.2337/dc22-2297 37134305 PMC10300520

[67] Johnson ML Bergenstal RM Levy BL Dreon DM . A safe and simple algorithm for adding and adjusting mealtime insulin to basal-only therapy. Clin Diabetes. (2022) 40:489–97. doi: 10.2337/cd21-0137 36381310 PMC9606561

[68] Di Giuseppe G Ciccarelli G Soldovieri L Capece U Cefalo CMA Moffa S . First-phase insulin secretion: can its evaluation direct therapeutic approaches? Trends Endocrinol Metab. (2023) 34:216–30. doi: 10.1016/j.tem.2023.02.001 36858875

[69] Lupsa BC Inzucchi SE . Use of SGLT2 inhibitors in type 2 diabetes: weighing the risks and benefits. Diabetologia. (2018) 61:2118–25. doi: 10.1007/s00125-018-4663-6 30132031

[70] Nauck MA Quast DR Wefers J Meier JJ . GLP-1 receptor agonists in the treatment of type 2 diabetes - state-of-the-art. Mol Metab. (2021) 46:101102. doi: 10.1016/j.molmet.2020.101102 33068776 PMC8085572

[71] Pullman J Darsow T Frias JP . Pramlintide in the management of insulin-using patients with type 2 and type 1 diabetes. Vasc Health Risk Manag. (2006) 2:203–12. doi: 10.2147/vhrm.2006.2.3.203 17326327 PMC1993989

[72] Moelands SV Lucassen PL Akkermans RP De Grauw WJ Van de Laar FA . Alpha-glucosidase inhibitors for prevention or delay of type 2 diabetes mellitus and its associated complications in people at increased risk of developing type 2 diabetes mellitus. Cochrane Database Syst Rev. (2018) 12:CD005061. doi: 10.1002/14651858.CD005061.pub3 30592787 PMC6517235

[73] Dalsgaard NB Gasbjerg LS Hansen LS Hansen NL Stensen S Hartmann B . The role of GLP-1 in the postprandial effects of acarbose in type 2 diabetes. Eur J Endocrinol. (2021) 184:383–94. doi: 10.1530/eje-20-1121 33449919

[74] Ferrannini E Ramos SJ Salsali A Tang W List JF . Dapagliflozin monotherapy in type 2 diabetic patients with inadequate glycemic control by diet and exercise: a randomized, double-blind, placebo-controlled, phase 3 trial. Diabetes Care. (2010) 33:2217–24. doi: 10.2337/dc10-0612 20566676 PMC2945163

[75] Stenlöf K Cefalu WT Kim KA Alba M Usiskin K Tong C . Efficacy and safety of canagliflozin monotherapy in subjects with type 2 diabetes mellitus inadequately controlled with diet and exercise. Diabetes Obes Metab. (2013) 15:372–82. doi: 10.1111/dom.12054 23279307 PMC3593184

[76] Prasad-Reddy L Isaacs D . A clinical review of GLP-1 receptor agonists: efficacy and safety in diabetes and beyond. Drugs Context. (2015) 4:212283. doi: 10.7573/dic.212283 26213556 PMC4509428

[77] Lyu B Hwang YJ Selvin E Jameson BC Chang AR Grams ME . Glucose-lowering agents and the risk of hypoglycemia: a real-world study. J Gen Intern Med. (2023) 38:107–14. doi: 10.1007/s11606-022-07726-8 35831767 PMC9849518

[78] Martens TW Bergenstal RM Pearson T Carlson AL Scheiner G Carlos C . Making sense of glucose metrics in diabetes: linkage between postprandial glucose (PPG), time in range (TIR) & hemoglobin A1c (A1C). Postgrad Med. (2021) 133:253–64. doi: 10.1080/00325481.2020.1851946 33315495

[79] Rafi E Tranchito L Hatipoglu B . Navigating insulin options for diabetes management. J Clin Endocrinol Metab. (2025) 110:S159–64. doi: 10.1210/clinem/dgae790 39998925

[80] Hope SV Jones AG Goodchild E Shepherd M Besser RE Shields B . Urinary C-peptide creatinine ratio detects absolute insulin deficiency in type 2 diabetes. Diabetes Med. (2013) 30:1342–8. doi: 10.1111/dme.12222 23659458 PMC4154136

[81] American Diabetes Association . 2. Diagnosis and classification of diabetes: Standards of Care in Diabetes-2026. Diabetes Care. (2026) 49:S27–49. doi: 10.2337/dc26-S002 41358893 PMC12690183

[82] Bluher M Malhotra A Bader G . Beta-cell function in treatment-naive patients with type 2 diabetes mellitus: analyses of baseline data from 15 clinical trials. Diabetes Obes Metab. (2023) 25:1403–7. doi: 10.1111/dom.14969 36594703

[83] Lane W Lambert E George J Rathor N Thalange N . Exploring the burden of mealtime insulin dosing in adults and children with type 1 diabetes. Clin Diabetes. (2021) 39:347–57. doi: 10.2337/cd20-0117 34866778 PMC8603317

[84] Candido R Wyne K Romoli E . A review of basal-bolus therapy using insulin glargine and insulin lispro in the management of diabetes mellitus. Diabetes Ther. (2018) 9:927–49. doi: 10.1007/s13300-018-0422-4 29654514 PMC5984925

[85] Girard J . The inhibitory effects of insulin on hepatic glucose production are both direct and indirect. Diabetes. (2006) 55:S65–9. doi: 10.2337/db06-S009

[86] American Diabetes Association . Insulin basics for diabetes. Available online at: https://diabetes.org/health-wellness/medication/insulin-basics (Accessed October 8, 2026).

[87] Kaiserman KB Christiansen M Bhavsar S Ulloa J Santogatta B Hanna J . Reduction in postprandial peak glucose with increased technosphere insulin dosage. J Diabetes Sci Technol. (2024) 18:397–401. doi: 10.1177/19322968221110622 35833638 PMC10973860

[88] Slattery D Amiel SA Choudhary P . Optimal prandial timing of bolus insulin in diabetes management: a review. Diabetes Med. (2018) 35:306–16. doi: 10.1111/dme.13525 29044708 PMC5836969

[89] Famulla S Hövelmann U Fischer A Coester H-V Hermanski L Kaltheuner M . Insulin injection into lipohypertrophic tissue: blunted and more variable insulin absorption and action and impaired postprandial glucose control. Diabetes Care. (2016) 39:1486–92. doi: 10.2337/dc16-0610 27411698

[90] Seaquist ER Blonde L McGill JB Heller SR Kendall DM Bumpass JB . Hypoglycaemia is reduced with use of inhaled Technosphere® insulin relative to insulin aspart in type 1 diabetes mellitus. Diabetes Med. (2020) 37:752–9. doi: 10.1111/dme.14202 31811662 PMC7216876

[91] Nathan DM Genuth S Lachin J Cleary P Crofford O Davis M . The effect of intensive treatment of diabetes on the development and progression of long-term complications in insulin-dependent diabetes mellitus. N Engl J Med. (1993) 329:977–86. doi: 10.1056/nejm199309303291401 8366922

[92] Heinemann L Baughman R Boss A Hompesch M . Pharmacokinetic and pharmacodynamic properties of a novel inhaled insulin. J Diabetes Sci Technol. (2017) 11:148–56. doi: 10.1177/1932296816658055 27378794 PMC5375067

[93] Black C Cummins E Royle P Philip S Waugh N . The clinical effectiveness and cost-effectiveness of inhaled insulin in diabetes mellitus: a systematic review and economic evaluation. Health Technol Assess. (2007) 11:1–126. doi: 10.3310/hta11330 17767897

[94] Gingras V Taleb N Roy-Fleming A Legault L Rabasa-Lhoret R . The challenges of achieving postprandial glucose control using closed-loop systems in patients with type 1 diabetes. Diabetes Obes Metab. (2018) 20:245–56. doi: 10.1111/dom.13052 28675686 PMC5810921

[95] Gerstein HC Miller ME Byington RP Goff DC Jr. Bigger JT Buse JB . Effects of intensive glucose lowering in type 2 diabetes. N Engl J Med. (2008) 358:2545–59. doi: 10.1056/NEJMoa0802743 18539917 PMC4551392

[96] Gubitosi-Klug RA Braffett BH White NH Sherwin RS Service FJ Lachin JM . Risk of severe hypoglycemia in type 1 diabetes over 30 years of follow-up in the DCCT/EDIC Study. Diabetes Care. (2017) 40:1010–6. doi: 10.2337/dc16-2723 28550194 PMC5521975

[97] Mathew P Thoppil D . Hypoglycemia. In: Statpearls (Internet), Treasure Island, FL, 2024.

[98] Przezak A Bielka W Molęda P . Fear of hypoglycemia-an underestimated problem. Brain Behav. (2022) 12:e2633. doi: 10.1002/brb3.2633 35620854 PMC9304823

[99] McGill JB Weiss D Grant M Jones MC Kendall DM Hoogwerf BJ . Understanding inhaled Technosphere Insulin: Results of an early randomized trial in type 1 diabetes mellitus. J Diabetes. (2021) 13:164–72. doi: 10.1111/1753-0407.13099 32737923

[100] Aschner P . New IDF clinical practice recommendations for managing type 2 diabetes in primary care. Diabetes Res Clin Pract. (2017) 132:169–70. doi: 10.1016/j.diabres.2017.09.002 28962686

[101] Hershon KS Hirsch BR Odugbesan O . Importance of postprandial glucose in relation to A1C and cardiovascular disease. Clin Diabetes. (2019) 37:250–9. doi: 10.2337/cd18-0040 31371856 PMC6640888

[102] Aryangat AV Gerich JE . Type 2 diabetes: postprandial hyperglycemia and increased cardiovascular risk. Vasc Health Risk Manag. (2010) 6:145–55. doi: 10.2147/vhrm.s8216 20448799 PMC2860446

[103] Hinnen DA . Therapeutic options for the management of postprandial glucose in patients with type 2 diabetes on basal insulin. Clin Diabetes. (2015) 33:175–80. doi: 10.2337/diaclin.33.4.175 26487791 PMC4608276

[104] Gao X Cai X Yang W Chen Y Han X Ji L . Meta-analysis and critical review on the efficacy and safety of alpha-glucosidase inhibitors in Asian and non-Asian populations. J Diabetes Investig. (2018) 9:321–31. doi: 10.1111/jdi.12711 28685995 PMC5835463

[105] Ryan G Briscoe TA Jobe L . Review of pramlintide as adjunctive therapy in treatment of type 1 and type 2 diabetes. Drug Des Devel Ther. (2009) 2:203–14. doi: 10.2147/dddt.s3225 19920907 PMC2761191

[106] Rehani PR Iftikhar H Nakajima M Tanaka T Jabbar Z Rehani RN . Safety and mode of action of diabetes medications in comparison with 5-aminolevulinic acid (5-ala). J Diabetes Res. (2019) 2019:4267357. doi: 10.1155/2019/4267357 31781665 PMC6874935

